# Enhancing the aluminium tolerance of barley by expressing the citrate transporter genes *SbMATE* and *FRD3*

**DOI:** 10.1093/jxb/eru121

**Published:** 2014-04-01

**Authors:** Gaofeng Zhou, Jorge F. Pereira, Emmanuel Delhaize, Meixue Zhou, Jurandir V. Magalhaes, Peter R. Ryan

**Affiliations:** ^1^Tasmanian Institute of Agriculture, University of Tasmania, PO Box 46, Kings Meadows, TAS 7249, Australia; ^2^CSIRO Plant Industry, GPO Box 1600, Canberra, ACT 2601, Australia; ^3^Embrapa Wheat, Rodovia BR 285 km 294, CEP 99001-970, Passo Fundo, RS, Brazil; ^4^Embrapa Maize and Sorghum, Rod. MG 424, Km 65, 35701-970, Sete Lagoas, Minas Gerais, Brazil

**Keywords:** Acid soil, cereal, citrate, *Hordeum vulgare*, MATE transporters, resistance, root exudates, transgenic.

## Abstract

Malate and citrate efflux from root apices is a mechanism of Al^3+^ tolerance in many plant species. Citrate efflux is facilitated by members of the MATE (multidrug and toxic compound exudation) family localized to the plasma membrane of root cells. Barley (*Hordeum vulgare*) is among the most Al^3+^-sensitive cereal species but the small genotypic variation in tolerance that is present is correlated with citrate efflux via a MATE transporter named HvAACT1. This study used a biotechnological approach to increase the Al^3+^ tolerance of barley by transforming it with two MATE genes that encode citrate transporters: *SbMATE* is the major Al^3+^-tolerance gene from sorghum whereas *FRD3* is involved with Fe nutrition in *Arabidopsis*. Independent transgenic and null T3 lines were generated for both transgenes. Lines expressing *SbMATE* showed Al^3+^-activated citrate efflux from root apices and greater tolerance to Al^3+^ toxicity than nulls in hydroponic and short-term soil trials. Transgenic lines expressing *FRD3* exhibited similar phenotypes except citrate release from roots occurred constitutively. The Al^3+^ tolerance of these lines was compared with previously generated transgenic barley lines overexpressing the endogenous *HvAACT1* gene and the *TaALMT1* gene from wheat. Barley lines expressing *TaALMT1* showed significantly greater Al^3+^ tolerance than all lines expressing *MATE* genes. This study highlights the relative efficacy of different organic anion transport proteins for increasing the Al^3+^ tolerance of an important crop species.

## Summary

Al^3+^ tolerance of barley was enhanced in transgenic plants by expression of the *SbMATE* gene from sorghum and the *FRD3* gene from *Arabidopsis*, which increased citrate efflux in roots.

## Introduction

The prevalence of toxic aluminium cations (Al^3+^) in acid soils (pH <5.0) is a major limitation to crop production around the world (Kochian *et al.*, 2004, 2005). Soluble Al^3+^ rapidly inhibits root growth by damaging interactions at the growing root apices (Ryan *et al.*, 1993; Sivaguru and Horst, 1998). This reduces the ability of roots to penetrate the soil and absorb water and nutrients. Some plant species have evolved mechanisms to combat this stress which either exclude Al^3+^ from the growing root apices or safely accommodate Al^3+^ once it enters the cytosol and efficiently repair stress-induced damage. One exclusion mechanism that has been described in a wide range of species relies on the release of organic anions, such as citrate and malate, from roots (Miyasaka *et al.*, 1991; Delhaize *et al.*, 1993; Ma *et al.*, 2001). These anions can form strong complexes with metal ions and it is hypothesized they protect cells at the growing root apex by chelating toxic Al^3+^ cations in the apoplasm and rhizosphere (Kinraide *et al.*, 2005).

The first gene identified in plants that was able to explain genotypic variation in Al^3+^ tolerance was *TaALMT1* (aluminium-activated anion transporter) from wheat (*Triticum aestivum*). *TaALMT1* encodes an Al^3+^-activated anion channel that facilitates malate efflux in roots (Sasaki *et al.*, 2004; Pineros *et al.*, 2008a; Zhang *et al.*, 2008). Other members of the ALMT family perform similar transport functions in *Arabidopsis thaliana* (Hoekenga *et al.*, 2006), *Brassica napus* (Ligaba *et al.*, 2006) and rye (*Secale cereale*) (Collins *et al.*, 2008). The release of citrate from roots is mediated by different transporters from the MATE (multidrug and toxic compound extrusion) family. The first *MATE* genes involved in Al^3+^ tolerance were identified by mapped-based cloning in barley (*Hordeum vulgare*) (Furukawa *et al.*, 2007) and sorghum (*Sorghum bicolor*) (Magalhaes *et al.*, 2007). The aluminium-activated citrate transporter gene (*HvAACT1*) in barley is constitutively expressed in the root apices, whereas *SbMATE* expression in sorghum is induced by Al^3+^ treatment over several days. However, in both cases, relative tolerance to Al^3+^ toxicity among different genotypes of barley and sorghum is highly correlated with the level of expression of these genes. Furthermore, these plants need to be exposed to Al^3+^ for citrate efflux to occur indicating that an interaction, either direct or indirect, between Al^3+^ and the MATE proteins is required to activate their function. Heterologous expression of *HvAACT1* in tobacco (*Nicotiana tabacum*) (Furukawa *et al.*, 2007) and *SbMATE* in an Al^3+^-sensitive mutant *Arabidopsis* line (Magalhaes *et al.*, 2007) increased the tolerance of these plants to Al^3+^ stress. Expression of *SbMATE* in wheat also increased the tolerance of transgenic T1 wheat lines but the phenotype proved unstable and was lost in subsequent generations (Magalhaes *et al.*, 2007; L.V. Kochian, personal communication). MATE genes were later linked with Al^3+^ tolerance in other species including *Arabidopsis*, maize (*Zea mays*), wheat, rice (*Oryza sativa*), and rice bean (*Vigna umbellata*) (Liu *et al.*, 2009; Ryan *et al.*, 2009, 2011; Yang *et al.*, 2011; Yokosho *et al.*, 2011; Maron *et al.*, 2013; Tovkach *et al.*, 2013). Most of these MATE proteins also require soluble Al^3+^ to activate their function by mechanisms that remain unclear. Two exceptions to this pattern include TaMATE1 in wheat and VuMATE1 from rice bean because both these proteins release citrate in the absence of Al^3+^. Interestingly, *VuMATE1* expression is still induced by Al^3+^ treatment (Yang *et al.*, 2011) whereas *TaMATE1* is expressed constitutively (Ryan *et al.*, 2009, 2011).

FRD3 is a member of the MATE family of proteins in *Arabidopsis* which transports citrate but not for Al^3+^ tolerance. Instead, FRD3 is expressed in the xylem paremchyma of roots where it transports citrate into the xylem to facilitate iron movement to the shoots in the transpiration stream (Durrett *et al.*, 2007). Nevertheless, when overexpressed in *Arabidopsis* with the 35SCaMV promoter, *FRD3* confers citrate efflux in roots and enhanced Al^3+^ tolerance compared to control plants. The FRD3 protein does not require Al^3+^ to activate its function so efflux in the roots of the transgenic *Arabidopsis* lines is constitutive. This contrasts with the MATE transporters from barley and sorghum, which are both activated by Al^3+^.

Although variation in *HvAACT1* expression in barley largely accounts for the genotypic variation in Al^3+^ tolerance, this species remains one of the most sensitive of agriculturally important grasses. Furthermore, there appears to be little potential for improvement in barley using conventional breeding methods beyond the levels currently provided by *HvAACT1* (Minella and Sorrells, 1992). Biotechnology has provided alternative strategies for increasing the basal tolerance of barley. Indeed, Al^3+^ tolerance has been improved in transgenic plants by increasing the expression of endogenous genes such as *HvAACT1* (Zhou *et al.*, 2013) and *HvALMT1* (Gruber *et al.*, 2011), and by heterologous expression of *TaALMT1* from wheat (Delhaize *et al.*, 2004) and the thioredoxin gene (*PTrx*) from *Phalaris coerulescensi* (Li *et al.*, 2010). Transgenic barley lines expressing *TaALMT1* also show improved phosphate uptake and grain yield when grown on an acid soil with low levels of plant-available phosphorus, which could largely be attributed to improved root growth (Delhaize *et al.*, 2009). There is no *a priori* reason to predict whether or not similar MATE genes from other species can confer even stronger phenotypes. Therefore, in the present study, barley was transformed with two MATE genes with contrasting characteristics to determine whether they could increase citrate release from roots and tolerance to Al^3+^ toxicity in hydroponics and in acid soil. The tolerance of these lines was compared with previously generated transgenic barely lines expressing the organic anion transporter proteins TaALMT1 and HvAACT1.

## Materials and Methods

### Plant materials and plasmid vectors

The Al^3+^-sensitive barley cv. Golden Promise was used in the transformation experiments and cv. Dayton was included as an Al^3+^-tolerant control. cDNAs for *SbMATE* and *FRD3* were inserted into the pWBVec8 binary vector (Wang *et al.*, 1998) where expression of the transgenes is driven by the maize ubiquitin promoter (Schunmann *et al.*, 2003). The pWBVec8::*SbMATE* and pWBVec8::*FRD3* vectors were introduced into *Agrobacterium* by triparental mating (Wise *et al.*, 2006).

### Barley transformation

Barley was transformed using the *Agrobacterium* method as described by Tingay *et al.* (1997). Primary transgenic plants (T0) transformed with *SbMATE* were analysed for the presence of the transgene using the following primers: forward 5′-GTCACCACGTCGTTCGTC-3′ and reverse 5′-GGGTGCAGATCTGGAAGG-3′. Four independent T1 transgenic lines (SbMATE:T1_9A, SbMATE:T1_22, SbMATE:T1_100E, and SbMATE:T1_133) exhibiting higher citrate efflux in root tips than wild-type plants were selected to generate T3 families, and from these putative homozygous and null lines were selected using PCR. Primary transgenic plants (T0) transformed with *FRD3* were tested for the presence of the transgene with the following primers: forward 5′-GCCCATGTCATTTCTCAGTACTTCA-3′ and reverse 5′-TTCCAAACTGCAAATCCCCGAAG-3′. Eight T1 lines were tested for citrate efflux, and two with the highest fluxes (FRD3:T1_40 and FRD3:T1_55) were selected to generate T3 families. Putative homozygous and null lines were selected for each using PCR.

### Quantitative reverse-transcription PCR

Three biological replicates each consisting of eight root apices (~5mm) were collected from seedlings and total RNA was extracted using a RNeasy Minikit (Qiagen) with DNAase treatment. First-strand cDNA was synthesized using 1 μg total RNA, 1× RT buffer, 10mM each dNTP, 500ng oligo(dT)_15_ primer, 0.2M dithiothreitol and 1 unit SuperScript II Reverse Transcriptase (Invitrogen). Reactions were incubated at 25 °C for 5min and then at 42 °C for 60min, followed by a RNaseH degradation step at 37 °C for 30min. Real-time PCR was performed in a Rotor-Gene 3000 Real Time Cycler (Corbett Research, Australia) using 10 μl reaction mixture containing 4.5 μl cDNA diluted to 1:20, 5 µl SYBR Green JumpStart Taq ReadyMix (Sigma), and 0.5 μl of 10 pmol μ l^–1^ each primer. The barley endogenous actin gene (forward: 5′-GACTCTGGTGATGGTGTCAGC-3′, reverse: 5′-GGCTGGAAGAGGACCTCAGG-3′) was used to normalize the transgene expression level. Primers used to measure *SbMATE* expression were 5′-ACCTGATAACGCTGATAATGCTGAG and 5′-CAGCAGAAGGAATCCGCATCC-3′ and for *FRD3* were 5′-GCCCATGTCATTTCTCAGTACTTCA-3′ and 5′-TTCCAAACTGCAAATCCCCGAAG-3′.

### Measurements of citrate and malate efflux

Citrate efflux from excised root apices was measured as described by Wang *et al.* (2007) and Zhou *et al* (2013). Seedlings were grown in nutrient solution without added AlCl_3_ for 4 d. Malate concentrations in samples were measured with an enzyme assay as described previously (Ryan *et al.*, 1995; Pereira *et al.*, 2010).

### Relative root length in hydroponic culture

A nutrient solution (pH 4.3) containing 500 μM KNO_3_, 500 μM CaCl_2_, 500 μM NH_4_NO_3_, 150 μM MgSO_4_, 10 μM KH_2_PO_4_, 2 μM Fe:EDTA, 11 μM H_3_BO_3_, 2 μM MnCl_2_, 0.35 μM ZnCl_2_, 0.2 μM CuCl_2_, and AlCl_3_ concentrations of 0, 1, 2, and 4 μM was prepared. The seeds were germinated in the dark for 2 d at 4 °C and 2 d at 24 °C. After the length of the middle primary root was measured, the seedlings were placed on floats in tanks each containing 20 l aerated nutrient solution with 0, 1, 2, and 4 μM AlCl_3_. After 4 d, the seedlings were removed and net root growth was calculated.

### Soil experiments

An acidic red ferrosol soil obtained from the Robertson region of New South Wales, Australia (34° 35′ S 150° 36′ E) was used in the soil experiment. The pH of half of the soil was raised from pH 4.33 to 5.18 (measured with 0.01M CaCl_2_) with addition of 5g CaCO_3_ kg^–1^ dry soil. This also reduced exchangeable Al^3+^ in the soil from approximately 30% of total exchangeable cations to below 1%. Each pot (diameter 9cm and height 22cm) contained 1.3kg soil. Field capacity of the soil was 36% and water was added to maintain moisture at 90% of the field capacity. No additional water was applied. Seeds from each line were germinated on Petri dishes and seedlings with similar root lengths were planted in three pots with acid soil and three pots with limed soil (two seedlings per pot). The pots were placed in a temperature-controlled glasshouse under a 16h/8h light/dark cycle (22 and 18 °C, respectively). After 6 d, the plants were harvested and shoot fresh weight obtained. Roots were washed using a gentle water spray and measurements made of the length of the longest two roots on each seedling. The whole root system was stored in 50% ethanol for later processing. Preserved roots were floated on a plastic tray and scanned using a flatbed scanner (Epson Expression 800) at a resolution of 400 dpi for total root length and diameter using WINRhizo Pro (version 2002). Roots were then dried at 70 °C for 48h and weighed.

### Statistical analysis

This study commonly compared root length in an Al^3+^ treatment (hydroponics or an acid soil) with root length in a control treatment (e.g. zero Al^3+^ in hydroponics or limed soil) to account for inherent differences in growth between lines. The resulting value is called relative root length (RRL). Therefore RRL = *x*/*y* where *x* and *y* represent the mean net root length in the Al^3+^ treatment and control conditions, respectively. Since standard errors (SE) are associated with the measurements of root length in the controls and treatments, the ratio of the means requires a new accumulated standard error. The formula for this accumulated error and the procedure used for determining whether two RRL values are statistically different from one another was described previously (Zhou *et al.*, 2013).

## Results

### Generation of T3 transgenic and null lines

**Fig. 1.**
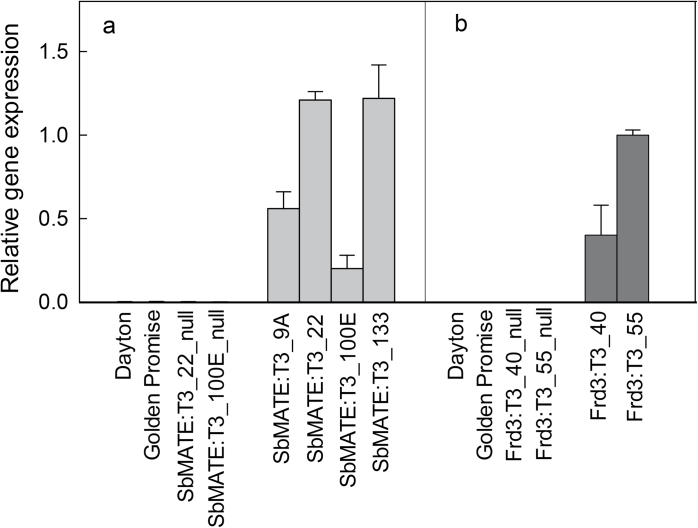
Relative expression of *SbMATE* (A) and *FRD3* (B) in transgenic and control barley lines. Quantitative reverse-transcription PCR was measured in T3 transgenic lines, null lines, and two wild-type control cultivars: Dayton (Al^3+^-resistant) and Golden Promise (Al^3+^-sensitive parental line). cDNA was prepared from root apices of barley lines and expression measured relative to the reference gene actin. Data are mean and standard error (*n*=3, biological replicates).

Barley (cv. Golden Promise) was transformed with the *SbMATE* gene from sorghum and the *FRD3* gene from *Arabidopsis*. Seedlings from independent T1 lines expressing these genes were grown hydroponically and citrate efflux measured in excised root apices in the presence of 50 μM AlCl_3_ (Supplementary Table S1 available at *JXB* online). Five of the seven *SbMATE* T1 lines tested showed significantly greater citrate efflux than the untransformed controls and four of these (SbMATE:T1_9A, SbMATE:T1_22, SbMATE:T1_100E, and SbMATE:T1_133) were used to generate T3 families. Among the T3 families, likely homozygous lines were identified for each transgenic event using PCR (Supplementary Table S2). Two null lines (untransformed segregants) were also identified from the SbMATE:T3_22 and SbMATE:T3_100E families and included as controls in later experiments along with the Al^3+^-sensitive parent cv. Golden Promise. Expression level in the root apices of the *SbMATE* T3 lines was measured with quantitative reverse-transcription PCR. All four transgenic lines had *SbMATE* expression in the roots, with SbMATE:T3_22 and SbMATE:T3_133 showing greater expression than the other two transgenic lines (Fig. 1A). No *SbMATE* expression was detected in the null lines, the parental cv. Golden Promise, and the Al^3+^-tolerant cv. Dayton.

Eight *FRD3* T1 lines were tested for citrate efflux and six showed greater efflux than controls (Supplementary Table S1). Two of these lines with the highest citrate efflux, FRD3:T1_40 and FRD3:T1_55, were used to generate T3 families, and likely T3 homozygous lines and null lines were identified from each transgenic event with PCR (Supplementary Table S3). The null lines FRD3:T3_40_null and FRD3:T3_55_null were included as controls in subsequent experiments. Expression of *FRD3* in FRD3:T3_55 was about 2.5-fold greater than in FRD3:T3_40. No *FRD3* expression was detected in the null lines or in the nontransgenic controls, as expected (Fig. 1B).

### Organic acid efflux in T3 lines

**Fig. 2.**
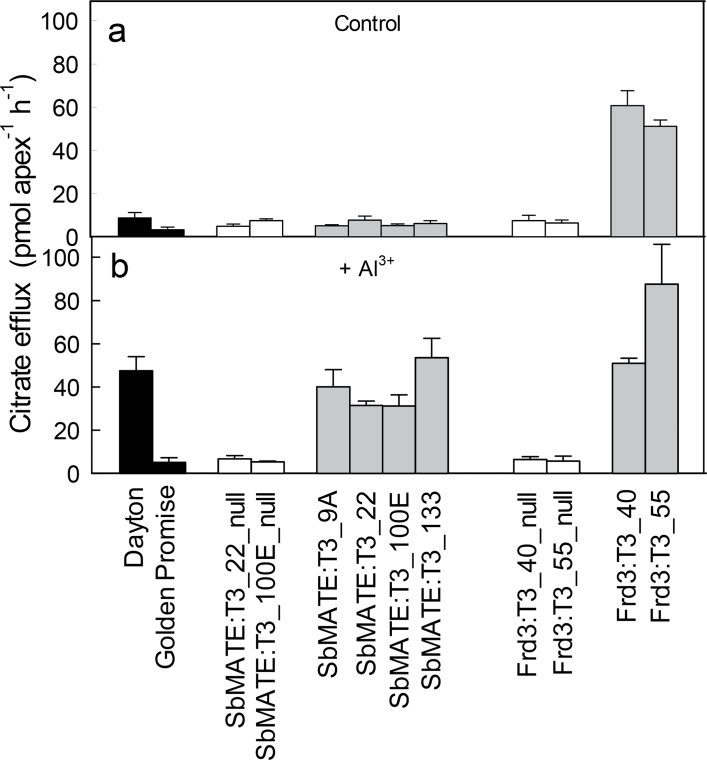
Citrate efflux from root apices. Citrate efflux from excised root apices of control lines and transgenic barley lines expressing *SbMATE* or *FRD3* in the absence of Al^3+^ (A) and in the presence of 50 μM AlCl_3_ (B). Black indicates the two wild-type controls, cv. Dayton (Al^3+^-resistant) and Golden Promise (Al^3+^-sensitive parental line); white indicates the two independent null segregant lines (T3) for the *SbMATE* and *FRD3* transformation events; grey indicates the independent T3 transgenic lines expressing the transgenes. Data are mean and standard error (*n*= 3 or 4),

Citrate efflux in the root apices of *SbMATE* and *FRD3* T3 lines were measured in the presence and absence of 50 μM AlCl_3_. In the absence of Al^3+^, citrate efflux from the *SbMATE* transgenic and null lines were low and similar to the untransformed controls which included the parental cv. Golden Promise and the Al^3+^-tolerant cv. Dayton (Fig. 2). By contrast, citrate efflux was detected in both *FRD3* transgenic lines in the absence of Al^3+^. In the presence of Al^3+^, citrate efflux was 40–80 pmol apex^–1^ h^–1^ from the transgenic lines expressing *SbMATE* and *FRD3* and in cv. Dayton. Only background efflux was measured in the null lines and cv. Golden Promise. Citrate efflux in cv. Dayton is controlled by the endogenous MATE gene *HvAACT1*. Wheat cv. Carazinho was also included as a positive control (data not shown) because it displays a high constitutive citrate efflux (Ryan *et al.*, 2009). Citrate efflux from Carazinho was about 2-fold greater than the barley lines, reaching 113±32 pmol apex^–1^ h^–1^ in the absence of Al^3+^ and 136±21 pmol apex^–1^ h^–1^ in the presence of 50 μM Al^3+^.

Malate efflux was also measured from root apices in the presence of 50 μM Al^3+^ but only low background fluxes were detected in the transgenic lines and control lines (data not shown). Malate efflux was detected in the wheat cv. Carazinho, which was included as a positive control. Efflux in Carazinho was 0.35 nmol apex^–1^ h^–1^ the presence of Al^3+^. This response is controlled by the TaALMT1 anion channel in wheat (Sasaki *et al.*, 2004).

### Al^3+^ tolerance: root growth in hydroponic experiments

**Fig. 3.**
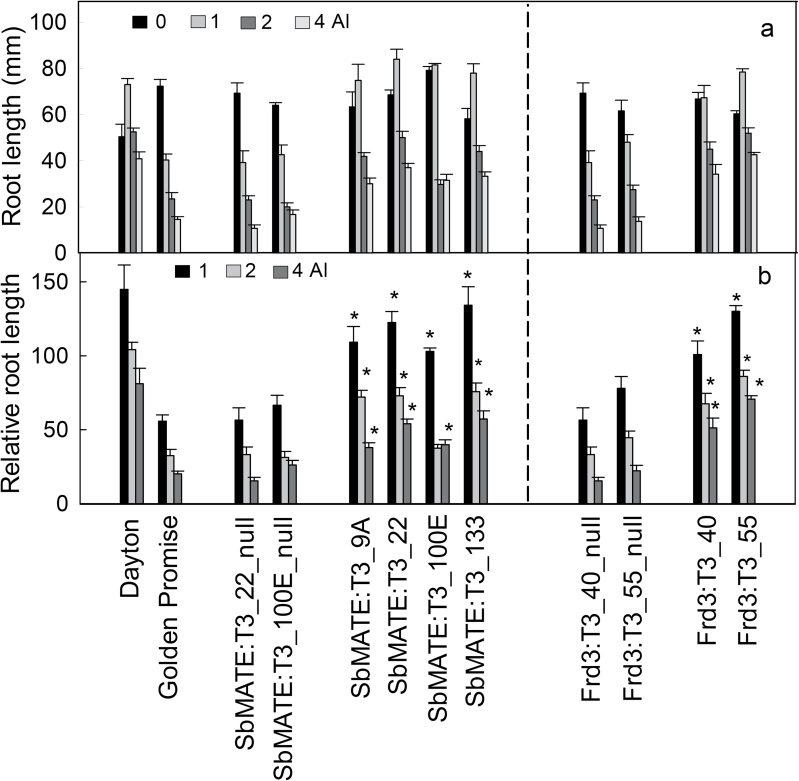
Al^3+^ tolerance of transgenic and control lines in hydroponic experiments. (A) Net root growth of transgenic and control seedlings was measured after 4 d in nutrient solution containing 0, 1, 2 or 4 μM AlCl_3_. Data are mean and standard error (*n*=7). (B) Relative root growth of lines was calculated as net root growth in Al^3+^ compared to zero Al^3+^ control. Data are means and standard error. Asterisks indicate significant differences from the null lines at the same Al^3+^ concentration (*P*<0.05).

After 4 d in hydroponic solution without Al^3+^, roots of all the lines grew 50–80mm (Fig. 3A). In the presence of 1 μM Al^3+^, growth of the null lines and cv. Golden Promise was inhibited by ~30%, whereas growth of the *SbMATE* and *FRD3* transgenic lines was either unaffected or stimulated. Increasing the Al^3+^ concentration to 2 or 4 μM inhibited root growth of all lines but the null lines and cv. Golden Promise were inhibited to a greater degree than either set of transgenic lines.

Relative root length compares net growth at each Al^3+^ concentration with the zero Al^3+^ treatment. As the Al^3+^ concentrations increased, RRL for the *SbMATE* transgenic lines and the *FRD3* transgenic lines remained 2–3-fold greater than their respective nulls. These differences were statistically significant (*P*<0.05) except for SbMATE:T3_100E, which was not different from its null lines at 2 μM Al^3+^ (Fig. 3B).

### Al^3+^ tolerance: root growth in soil experiments

**Fig. 4.**
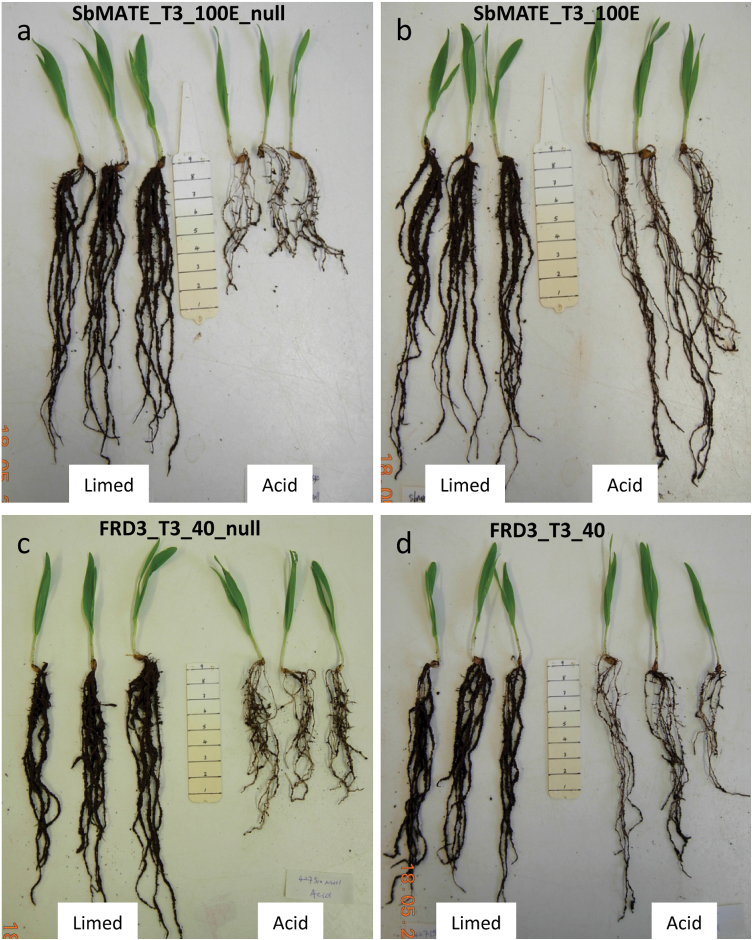
Barley plants at the end of the soil experiment. Representative plants of SbMATE:T3_100E_null (A), SbMATE:T3_100E (B), FRD3:T3_40_null (C), and FRD3:T3_40 (D) grown in limed and acid soil. Note that the roots on the transgenic plants and null plants are similar in the limed soil but longer on the transgenic plants than the null controls but in acid soil. Shoots do not show strong phenotypes between the acid and limed soils (this figure is available in colour at *JXB* online).

Soil experiments were performed over 6 d with an acidic ferrosol and the same soil amended with lime to increase the pH and decrease Al^3+^ toxicity. Root and shoot measurements were expressed as relative values from the acid soil compared to the limed soil. Representative plants immediately after harvest are shown in Fig. 4. Root fresh weight was similar or slightly greater in the acid soil compared to the limed soil for all lines except SbMATE:T3_100E_null, which was significantly lower in the acid soil (Supplementary Table S4). However, there were no consistent trends in the relative fresh weight of roots or shoots (data not shown) between the transgenic lines and their null controls for either of the *SbMATE* and *FRD3* lines.

**Fig. 5.**
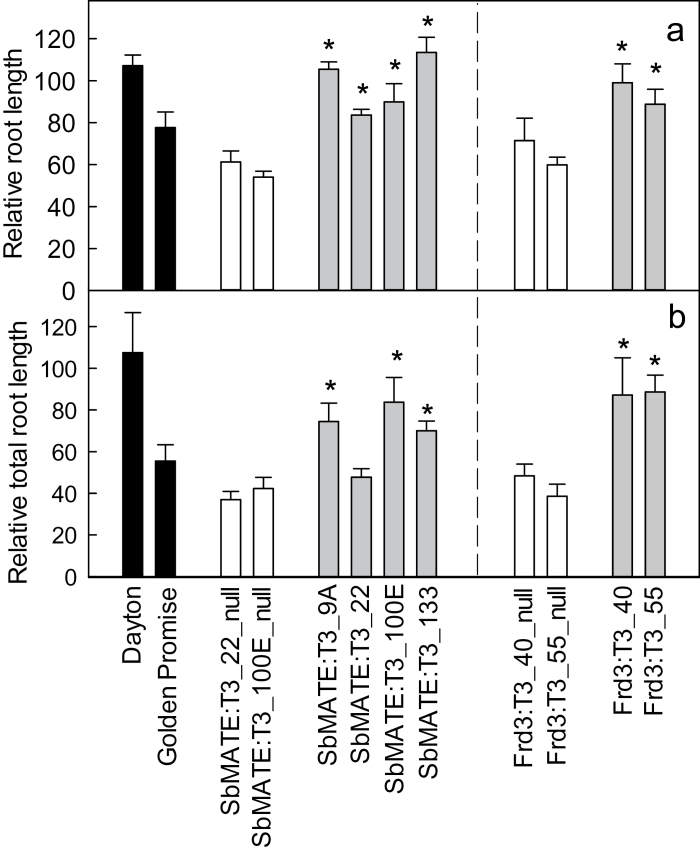
Al^3+^ tolerance in soil experiment: relative root lengths. (A) Relative root length based on the length of the longest root from seedlings grown in acid soil compared to the limed soil; data are mean and standard error; asterisks indicate significant differences from the null lines (*P*<0.05). (B) Relative total root growth of lines based on root length in acid soil compared to the limed soil; data are mean and standard error; asterisks above the *SbMATE* transgenic lines indicate significant differences from both null lines (*P*<0.05). Asterisks above the *FRD3* transgenic lines indicate significant differences from their respective null lines (*P*<0.05). See Supplementary Fig. S1 for raw data used to calculate these results.

Length of the longest root on seedlings in limed soil ranged from 160 to 210mm (Supplementary Fig. S1A). Roots were shorter in acid soil for both sets of null lines, with RRL approximately 60–70% (Fig. 5A). By contrast, RRL for the *SbMATE* transgenic lines was 85–115% and for the two *FRD3* transgenic lines RRL was 90 and 100%. Root growth of cv. Dayton was the same in the acid and limed soil. RRL for cv. Golden Promise was greater than both *SbMATE* null lines and one of the *FRD3* null lines in this experiment.

Total root length in both sets of null lines was ~60% less in acid soil compared to the limed soil (Supplementary Fig. S1B). Relative total root length in three of the four *SbMATE* transgenic lines and both *FRD3* transgenic lines was 2-fold greater than their respective nulls (Fig. 5B). The single transgenic line not fitting this trend was SbMATE:T3_22, which showed a larger-than-expected decrease in root growth in acid soil. Relative total root length in cv. Dayton was similar in acid and limed soils.

**Fig. 6.**
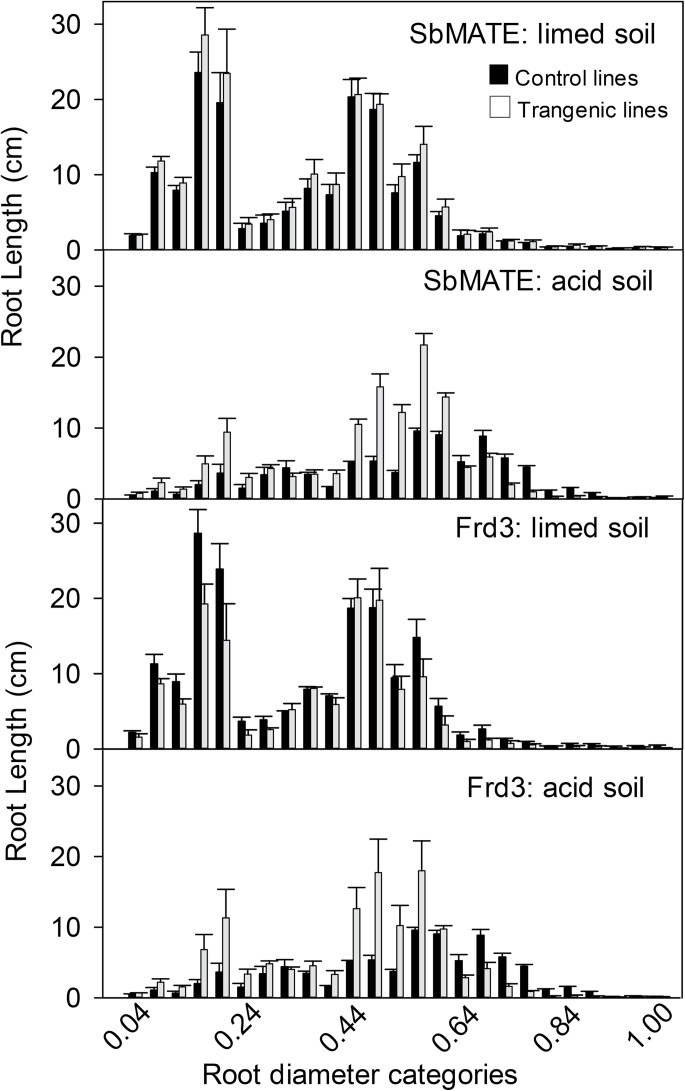
Distribution of root diameters on soil-grown plants. Transgenic plants expressing *SbMATE* or *FRD3* and control plants were grown in a limed soil or acid soil for 6 d. The roots were washed and scanned with WinRHIZO and the total root length falling within each diameter class was estimated. Black indicates control lines not expressing a transgene and these include the two nulls and Golden Promise (mean and standard error, *n*=3); grey indicates transgenic lines expressing a transgene; data are mean and standard error for the *SbMATE* lines (*n*=4) and mean and range for *FRD3* lines (*n*=2). The diameter categories increase in 40-μm increments from 0 to 1.0mm.

The combined root length within each category of root diameter was plotted for the transgenic and null lines (Fig. 6). For clarity, data from the independent null lines were combined and compared with the combined data from the transgenic lines. In limed soil, the distribution of root diameters was qualitatively similar for all the null and transgenic lines with two prominent peaks emerging at about 0.2 and 0.5mm (Fig. 6A, C). In acid soil, the distribution of diameters from Golden Promise and null lines of *SbMATE* (Fig. 6B) and *FRD3* (Fig. 6D) was significantly flatter than the limed soil with no peak close to 0.2mm, indicating proportionally more roots had diameters 0.6mm and greater. Transgenic lines expressing *SbMATE* and *FRD3* also showed a flatter profile and a shift towards thicker roots; however, the changes were not as large and the two distinct peaks close to 0.2 and 0.5mm remained. These results indicate that expression of single gene in barley enables roots to grow longer in an acid soil and maintains a greater proportion of thinner roots.

### Comparing transgenic barley lines transformed with MATE and ALMT genes

Previous studies have improved the Al^3+^ tolerance of barley by transforming the sensitive cv. Golden Promise with MATE genes and with ALMT genes. The present work directly compared the transgenic lines generated here with transgenic lines generated previously. The first experiment compared lines expressing *FRD3* and *SbMATE* with lines overexpressing the endogenous *HvAACT1* gene from barley (Zhou *et al.*, 2013). Plants were grown in hydroponics with 0, 1, 2, and 4 μM AlCl_3_ and relative root growth was estimated after 4 d. The tolerance conferred by the three MATE genes was similar for 1 and 2 μM AlCl_3_ but the *FRD3* line was significantly more tolerant than the other two lines at 4 μM (Supplementary Fig. S2).

**Fig. 7.**
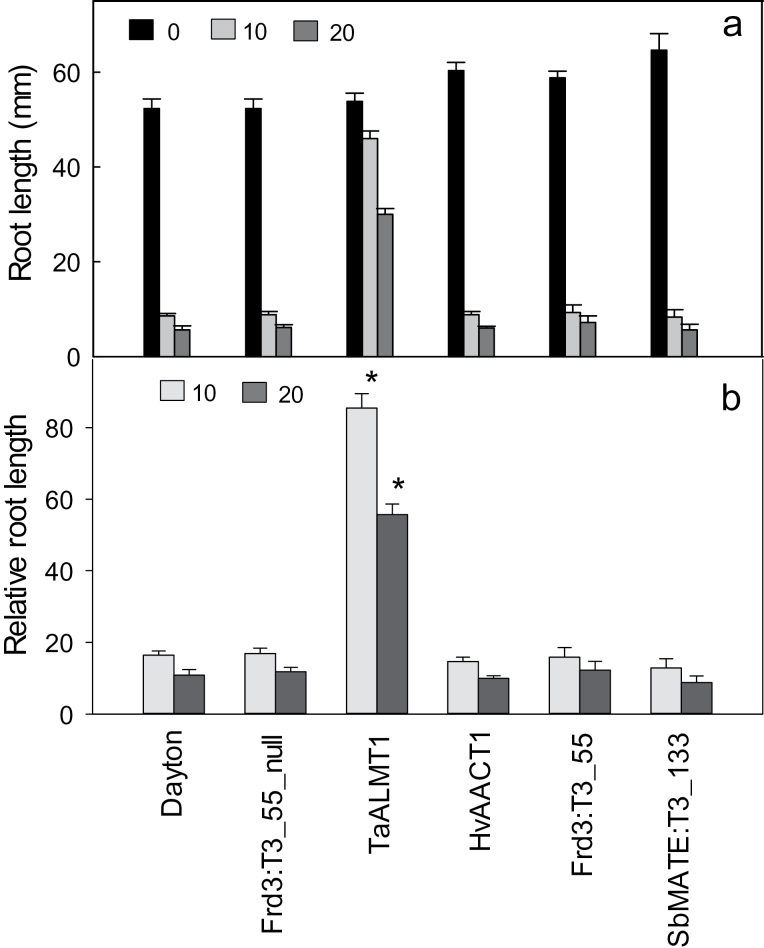
Direct comparison of Al^3+^ tolerance in transgenic barley lines expressing MATE genes and *TaALMT1*. T3 barley lines expressing *SbMATE* and *FRD3* (generated here) and *HvAACT1* (generated previously; Zhou *et al.*, 2013) were compared with a transgenic line expressing *TaALMT1* from wheat (Delhaize *et al.*, 2004). (A) Net root length was measured after plants were grown for four d in hydroponics with 0, 10 and 20 μM AlCl_3_. (B) Relative root length of lines was calculated as net root growth in Al^3+^ compared to zero Al^3+^ control. Data are mean and standard error (*n*= 5 or 6). Asterisks indicate significant differences from all other lines in the same condition (*P*<0.05).

In separate experiments, barley lines expressing the same three MATE genes were compared with barley expressing *TaALMT1* from wheat (Delhaize *et al.*, 2004). These experiments used higher concentrations of AlCl_3_ because preliminary experiments indicated they differentiated these lines more clearly. The results indicate that expression of the wheat gene *TaALMT1* conferred significantly greater tolerance at 10 and 20 μM Al^3+^ than any of the three *MATE* genes tested (Fig. 7).

## Discussion

In this study, barley was transformed with two MATE genes that encode citrate transporters with distinct properties. SbMATE is an Al^3+^-activated transport protein that confers Al^3+^ tolerance to sorghum (Magalhaes *et al.*, 2007) and FRD3 is involved in the long-distance transport of Fe from roots to shoots in *Arabidopsis* (Durrett *et al.*, 2007). Independent transgenic lines expressing these genes were generated and their Al^3+^ tolerance were compared with null lines. Transgenic lines expressing *SbMATE* displayed an Al^3+^-dependent citrate release from roots not detected in null segregant lines. These lines also showed greater tolerance to Al^3+^ and maintained a higher proportion of thinner roots, which is important for soil exploration and nutrient uptake. Barley lines expressing *FRD3* showed similar phenotypes except that citrate release occurred in the presence and absence of Al^3+^. These phenotypes are consistent with previous results from transgenic *Arabidopsis* expressing *FRD3* with the 35SCaMV promoter (Durrett *et al.*, 2007) and they confirm that, when expressed ectopically, *FRD3* can facilitate citrate efflux from monocotyledons in the absence of Al^3+^. The Al^3+^-tolerant barley cv. Dayton also showed citrate efflux but only in the presence of Al^3+^, which is consistent with previous findings (Furukawa *et al.*, 2007; Wang *et al.*, 2007).

Al^3+^ tolerance was evaluated in hydroponic experiments and in acid soil. Fresh shoot weight and fresh root weight were not strongly correlated with Al^3+^ tolerance which is similar to previous reports for young seedlings (Muhling *et al.*, 1988; Carr and Ritchie, 1993). Relative root length in the short-term hydroponics and soil trials proved to be a convenient screening method for comparing lines. At low Al^3+^ concentrations, both sets of transgenic lines and cv. Dayton had longer roots than in zero Al^3+^ solution. This stimulation of growth in acid conditions by low levels of Al^3+^ has been observed previously and is interpreted as Al^3+^ alleviating H^+^ toxicity (Kinraide *et al.*, 1992). Moroni *et al.* (2010) screened a range of cereal genotypes for Al^3+^ tolerance and concluded that the rankings differed between hydroponic and field trials because barley appeared more tolerant in soil than the hydroponic screens. While the present study found broad agreement between the results in hydroponics and short soil experiments, longer field trials will be necessary to assess how effective these transgenes are in improving grain yield on acid soils. In soil experiments, cv. Golden Promise did not always perform the same as the null lines even though the lines should have been genetically identical (Fig. 5). This is not unexpected since, unlike cv. Golden Promise, the nulls were regenerated from callus in tissue culture, which might have resulted in somaclonal variation. This highlights the importance of including null-segregant lines as controls when evaluating transgenic material.

Barley lines expressing *SbMATE* and *FRD3* showed similar levels of tolerance and none were more tolerant than the wild-type cv. Dayton. Interestingly citrate efflux in independent transgenic lines expressing *SbMATE* was similar despite differences in expression level (Figs 1 and 2). This result suggests that beyond a certain level of expression, citrate efflux, and consequently Al^3+^ tolerance, does not increase further. It is unclear why this saturation in tolerance occurs. Perhaps once the capacity to release citrate from root cells reaches a certain threshold, other metabolic processes begin to limit efflux, or the citrate efflux in nontargeted tissues becomes counterproductive to growth. Organic anion synthesis might limit citrate efflux when expression of the transporters is sufficiently high. Consistent with this idea are the findings that overexpression of genes involved in organic anion synthesis can also enhance Al^3+^ tolerance by increasing organic anion efflux in roots (Fuente *et al.*, 1997; Koyama *et al.*, 2000; Tesfaye *et al.*, 2001; Anoop *et al.*, 2003; Barone *et al.*, 2008; Trejo-Tellez *et al.*, 2010; Wang *et al.*, 2010). The present study found that a barley line transformed with *TaALMT1* from wheat (conferring malate efflux) was more tolerant than any of the lines expressing *MATE* genes. The efflux of citrate in lines expressing the *MATE* genes (~50 pmol apex^–1^ h^–1^) was approximately 20-fold smaller than malate efflux in lines expressing *TaALMT1* (~1 nmol apex^–1^ h^–1^). The stability constants for aluminium citrate compounds are many orders of magnitude greater than for aluminium malate compounds (Jones, 1998) suggesting that citrate should provide much greater tolerance than malate, even if less is released. This is partly supported by predictions of the chemical speciation program GEOCHEM (Shaff *et al.*, 2010). In a test solution of 1mM CaCl_2_, 100 μM AlCl_3_, and either 100 μM citrate or malate (fixed pH 4.5), the free Al concentration was 9.9 μM for malate and 0.07 μM for citrate. However, when 10-fold less malate and citrate are tested (ie. 10 μM citrate), the free concentration of Al was ~70 μM for both. Therefore the difference between these anions appears to depend on the ratio of their concentrations to Al. The relative toxicity of Al solutions to plants are best predicted by modelling the activities of the Al^3+^ species at the surface of the root cell membranes but this is a more complex calculation requiring some knowledge of the zeta potential or surface charge density of the roots (Kinraide *et al.*, 1992, 2005). The present finding that citrate efflux was not as effective as expected (Zhao *et al.*, 2003) might also be explained, in part, by inappropriate stability constants due to the experimental conditions used to derive these values. High concentrations of reagents are commonly used in high-ionic-strength background solutions which contrasts with the low ionic strength of the hydroponic growth solution. Furthermore, the theoretical stability constants derived for Al^3+^ and citrate might be difficult to interpret: at pH 4.3, the molar fraction of the trivalent citrate is relatively small. Nevertheless, the results appear to indicate that the stability constants for Al^3+^:citrate and Al^3+^:malate are less important than the magnitudes of fluxes. As the organic anions are released from the root-cell cytosol into the more acidic apoplastic environment, they will bind with H^+^ and potentially raise the pH slightly near the membrane surface; therefore, it is possible that differences in the capacity of these anions to influence local pH could also contribute to the tolerance they confer.

The finding that Al^3+^ tolerance was increased by *FRD3* expression, a gene not naturally involved with this phenotype, is consistent with a hypothesis for the evolution of Al^3+^ tolerance in plants (Magalhaes, 2010; Ryan and Delhaize, 2010). It proposes that Al^3+^ tolerance in some species is a relatively recent trait acquired from mutations to genes encoding organic anion transporters that perform other functions. These mutations affect the level or distribution of protein expression which extends their function to include organic anion release from root apices. This hypothesis is supported by reports describing how different mutations upstream of organic anion transporter genes change expression and alter responses to Al^3+^ stress. For example, multiple, perfect, tandem repeats of sequence in the promoter of *TaALMT1* in wheat (Sasaki *et al.*, 2006, Ryan *et al.*, 2009) and *cis*-acting elements in the promoter of *HlALMT1* in *Holcus lantanus* drive higher expression of these genes. Similarly, higher expression of *SbMATE* in different genotypes of sorghum is associated with a greater numbers of Tourist-like miniature inverted-repeat transposable elements (MITE) several kilobases upstream from the coding region (Magalhaes *et al.*, 2007). Other mutations that increase gene expression include transposon-like insertions in the 5′-untranscribed region of *HvAACT1* in barley (Fujii *et al.*, 2012) and in *TaMATE1B* in wheat (Tovkach *et al.*, 2013).

In conclusion, these experiments demonstrate that heterologous expression of *SbMATE* and *FRD3* can stably increase the Al^3+^ tolerance of an important cereal species by enhancing citrate efflux in root apices. Future studies will introgress these transgenes into Al^3+^-tolerant barley cultivars such as Dayton and perhaps pyramid them with other genes to assess whether the effects of the endogenous and transgenes are additive. Pyramiding *MATE* genes with *ALMT* genes is likely to be a more successful strategy than pyramiding multiple *MATE* genes. Since MATEs release citrate and ALMTs release malate, their combination could avoid the saturation of tolerance observed here when *MATE* genes were overexpressed. However overexpression of *MATE* or *ALMT* genes could have pleiotropic effects on plants through interactions with cell signalling or through chemical changes in the rhizosphere. For instance, some ALMTs are involved in mineral nutrition and ion homeostasis and so these processes could be perturbed in transgenic plants (Pineros *et al.*, 2008b; Gruber *et al.*, 2011). Malate release from *Arabidopsis* roots via AtALMT1 can induce the colonization of microorganisms in the rhizosphere and on the root surface (Rudrappa *et al.*, 2008). Malate exudation was shown to recruit *Bacillus subtilis* biofilm formation on tomato roots in a similar way (Chen *et al.*, 2012). Therefore, the root microbiome could be altered in transgenic plants with higher expression of MATE and ALMT transporters. It was recently shown that *AtALMT1* expression in *Arabidopsis* was increased by indole acetic acid, abscisic acid, and the bacterial elicitor flagellin 22 as well as to low pH and Al^3+^, which indicates this gene is potentially involved in a wide array of biological functions in addition to Al^3+^ tolerance (Kobayashi *et al.*, 2013). Therefore cell signalling, growth, or responses to stress could also be affected in plants overexpressing these transporters. The transgenic lines generated here and previously provide useful material for investigating these processes in more detail.

## Supplementary material

Supplementary data are available at *JXB* online.

Supplementary Fig. S1. Al^3+^ tolerance in soil experiments: total root length.

Supplementary Fig. S2. Comparing the Al^3+^ tolerance of barley lines expressing three different MATE genes.

Supplementary Table S1. Citrate efflux in the excised root apices of T1 transgenic lines.

Supplementary Table S2. Screening T2 barley plants transformed with *SbMATE*.

Supplementary Table S3. Screening T2 barley plants transformed with *FRD3*.

Supplementary Table S4. Root fresh weight of transgenic and control barley lines grown in acid and limed soil.

Supplementary Data
