# Supplementary material for: Enhancing the aluminium tolerance of barley by expressing the citrate transporter genes SbMATE and FRD3
Source: J Exp Bot. 2014 Apr 1;65(9):2381–90. doi: 10.1093/jxb/eru121 (PMC4036506; doi:10.1093/jxb/eru121)
Supplement: Supplementary Data [file supp_65_9_2381__index.html]

Enhancing the aluminium tolerance of barley by expressing the citrate transporter genes SbMATE and FRD3 — Enhancing the aluminium tolerance of barley by expressing the citrate transporter genes SbMATE and FRD3 — Supplementary Data 

# Enhancing the aluminium tolerance of barley by expressing the citrate transporter genes *SbMATE* and *FRD3*

## Supplementary Data

Data files

**Files in this Data Supplement:**

- Supplementary Data - Supplementary Data
