# Supplementary material for: Enhancing the aluminium tolerance of barley by expressing the citrate transporter genes SbMATE and FRD3
Source: J Exp Bot. 2014 Apr 1;65(9):2381–90. doi: 10.1093/jxb/eru121 (PMC4036506; doi:10.1093/jxb/eru121)
Supplement: Supplementary Data [file supp_eru121_jexbot116657_file001.pdf]

SUPPLEMENTARY MATERIAL

Enhancing the aluminium tolerance of barley by expressing the citrate transporter genes *SbMATE* and *FRD3*

Gaofeng Zhou, Jorge F. Pereira, Emmanuel Delhaize, Meixue Zhou, Jurandir V Magalhaes, Peter R. Ryan

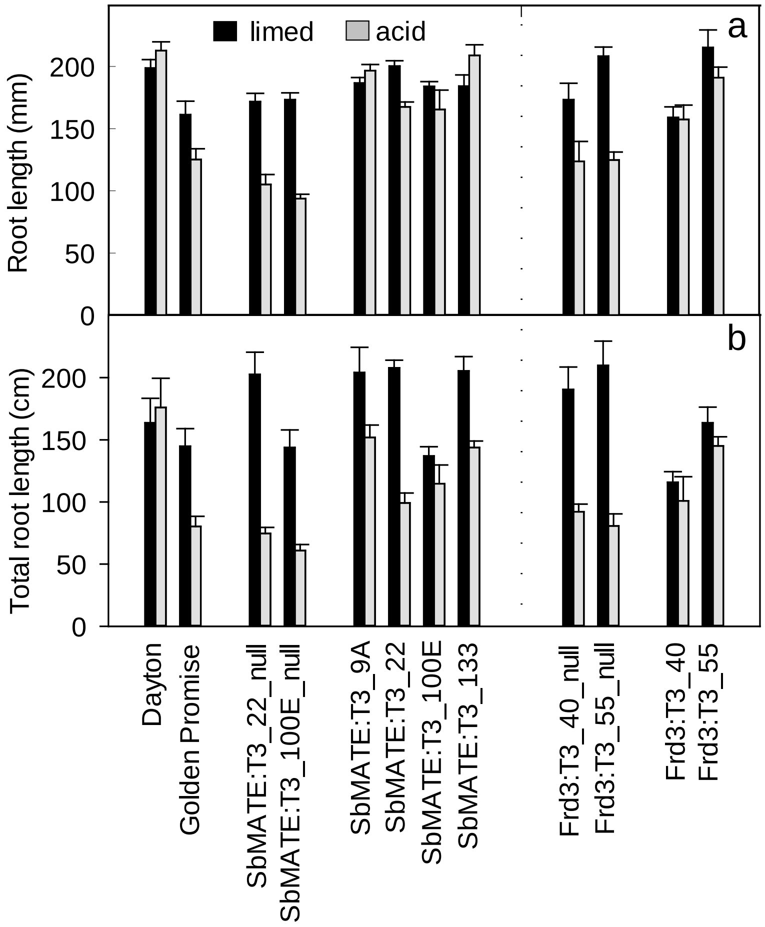

Figure S1 Al^{3+} tolerance in soil experiments: Root length data.
(a) Length of the longest roots on transgenic and control seedlings measured after six days growth in an acid and limed soil. Data show mean and standard error (n=6) (b) Total root length in transgenic and control seedlings was measured after six days in an acid and limed soil. Data show mean and standard error (n=6) Data show means and standard error.

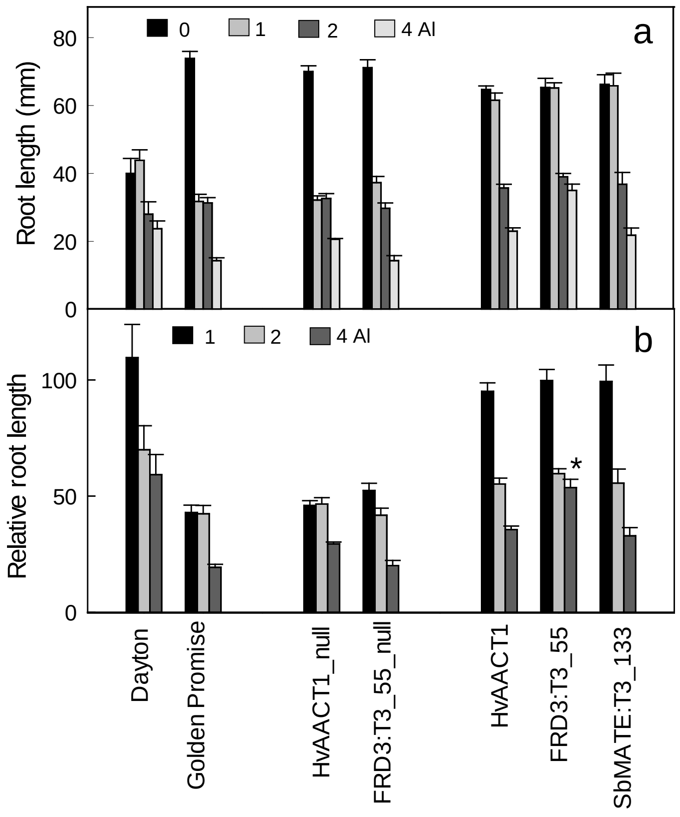

Figure S2 Comparing the Al^{3+} tolerance of barley lines expressing three different *MATE* genes.

The net root length (a) and relative root length (b) of transgenic barley lines expressing *Frd3* and *SbMATE* were compared with a line expressing *HvAACT1* generated previously (Zhou et al. 2013). Plants were grown for four days in hydroponics with 0, 1, 2, and 4 µM $AlCl_3$. Data show mean and standard error (n=6). Relative root growth among the three transgenic barley lines was the same except for FRD3:T3_55 at 4 µM $AlCl_3$ which was greater than the others.

Table S1. Citrate efflux from the excised root apices of T1 transgenic lines expressing SbMATE and FRD3 as indicated. Mean and standard error (n=3). Asterisk indicates fluxes greater than Golden Promise (P <0.05) using a t test.

Lines	Citrate efflux (pmol $apex^{-1}h^{-1}$)
Golden Promise	10.5±3.3
SbMATE:T1_9A	26.5±4.3*
SbMATE:T1_22	25.9±1.9*
SbMATE:T1_39	12.3±0.8
SbMATE:T1_98	18.8±4.5
SbMATE:T1_100E	33.0±1.7*
SbMATE:T1_119	25.6±4.6*
SbMATE:T1_133	36.7±1.3*
Golden Promise	4.6±1.5
FRD3:T1_38A	6.4±1.0
FRD3:T1_40	16.2±3.0*
FRD3:T1_42	11.6±3.0*
FRD3:T1_49	11.0±1.0*
FRD3:T1_52A	6.4±1.0
FRD3:T1_55	14.5±3.0*
FRD3:T1_58A	10.4±1.0*
FRD3:T1_83	10.4±2.5*

Table S2 Screening T2 barley plants transformed with *SbMATE* to identify putative homozygous lines and null lines. Presence of the transgene in genomic DNA was identified by PCR and the following primers: forward primer 5’-GTCACCACGTCGTTCGTC-3’ and reverse primer 5’-GGGTGCAGATCTGGAAGG-3’.

Lines	Transgenic	Non-transgenic	Total	Chosen
**Line T2_9A**				
T2_9A-1	14	5	19	
T2_9A-2	13	9	22	
T2_9A-3	3	9	12	
**T2_9A-5**	**19**	**0**	**19**	**Homozygous**
T2_9A-7	2	4	6	
**Line T2_22**				
T2_22-1	12	4	16	
**T2_22-2**	**0**	**20**	**20**	**Null**
T2_22-3	13	5	18	
T2_22-4	17	4	21	
T2_22-6	0	12	12	
**T2_22-7**	**19**	**0**	**19**	**Homozygous**
T2_22-8	15	0	15	
T2-22-10	3	2	5	
**Line T2_100E**				
**T2_100E-1**	**21**	**0**	**21**	**Homozygous**
T2_100E-2	14	5	19	
T2_100E-3	3	2	5	
**T2_100E-4**	**0**	**22**	**22**	**Null**
**Line T2_133**				
T2_133-3	2	0	2	
T2_133-6	3	0	3	
T2_133-7	2	1	3	
T2_133-8	10	4	14	
**T2_133-9**	**26**	**0**	**26**	**Homozygous**

Table S3 Screening T2 barley plants transformed with *FRD3* to identify putative homozygous lines and null lines. Presence of the transgene in genomic DNA was identified by PCR and the following primers: forward 5'-GCCCATGTCATTTCTCAGTACTTCA-3' and reverse 5'-TTCCAAACTGCAAATCCCCGAAG-3'.

Lines	Transgenic	Non-transgenic	Total	Selected
**Line T2_40**				
T2_40-1	2	2	4	
T2_40-2	1	0	1	
T2_40-3	12	0	12	
T2_40-4	5	1	6	
T2_40-5	13	2	15	
T2_40-6	14	3	17	
T2_**40-7**	**11**	**0**	**11**	**Homozygous**
T2_40-8	2	0	2	
T2_40-9	9	0	9	
T2_40-10	5	1	6	
T2_**40-11**	**0**	**21**	**21**	**Null**
**Line T2_55**				
T2_**55-1**	**0**	**21**	**21**	**Null**
T2_55-2	14	0	14	
T2_**55-3**	**9**	**0**	**9**	**Homozygous**
T2_55-4	12	6	18	
T2_55-5	7	3	10	
T2_55-6	17	6	23	
T2_55-7	7	0	7	
T2_55-8	4	3	7	
T2_55-9	0	9	9	
T2_55-10	3	0	3	

Table S4 Root fresh weight of transgenic and control barley lines grown in acid and limed soil from Figures 4 and 5. Relative root fresh weight is weight from the acid soil relative to the limed soil. Data show mean and SE (n=6).

Lines	Limed soil Root weight (g)	Acid soil Root weight (g)	Relative root weight (%)
Dayton	0.176±0.017	0.222±0.014	126 ± 14
Golden Promise	0.171±0.017	0.205±0.017	120 ± 16
SbMATE:T3_22_null	0.177±0.008	0.178±0.016	101 ± 10
SbMATE:T3_100E_null	0.192±0.017	0.130±0.009	68 ± 8
SbMATE:T3_9A	0.213±0.014	0.259±0.017	122 ± 11
SbMATE:T3_22	0.177±0.006	0.184±0.013	104 ± 8
SbMATE:T3_100E	0.194±0.010	0.211±0.015	109 ± 10
SbMATE:T3_133	0.218±0.010	0.236±0.016	108 ± 9
FRD3:T3_40_null	0.195±0.011	0.206±0.012	106 ± 8
FRD3:T3_55_null	0.216±0.010	0.202±0.014	93 ± 8
FRD3:T3_40	0.137±0.010	0.170±0.023	124 ± 19
FRD3:T3_55	0.207±0.016	0.233±0.010	113 ± 10
